# Demonstrating soft X-ray tomography in the lab for correlative cryogenic biological imaging using X-rays and light microscopy

**DOI:** 10.1038/s41598-025-29385-5

**Published:** 2025-11-27

**Authors:** Stephen O’Connor, David Rogers, Maryna Kobylynska, James A. Geraets, Katja Thaysen, Jacob Marcus Egebjerg, Madeleen C. Brink, Louisa Herbsleb, Michaela Salakova, Leon Fuchs, Frauke Alves, Claus Feldmann, Axel Ekman, Paul Sheridan, William Fyans, Tony McEnroe, Fergal O’Reilly, Kenneth Fahy, Roland A. Fleck, Daniel Wüstner, Jeremy C. Simpson, Andreas Walter, Sergey Kapishnikov

**Affiliations:** 1 SiriusXT, 9A Holly Ave., Stillorgan Industrial Park, Blackrock, Co. Dublin, Ireland; 2 Randall Centre for Cell and Molecular Biophysics, King’s College London, London, SE1 1UL UK; 3 Centre for Ultrastructural Imaging, King’s College London, London, SE1 1UL UK; 4 Department of Biochemistry and Molecular Biology, University of Southern Denmark, Odense M, Denmark; 5 Cell Screening Laboratory, School of Biology and Environmental Science, University College Dublin, Belfield, Dublin 4, Ireland; 6 Center of Optical Technologies, Aalen University, Aalen, Germany; 7 Translational Molecular Imaging, Max-Planck-Institute of Multidisciplinary Sciences, Göttingen, Germany; 8 Institute of Inorganic Chemistry, Karlsruhe Institute of Technology (KIT), Karlsruhe, Germany; 9 Department of Biological and Environmental Science and Nanoscience Centre, University of Jyväskylä, Jyväskylä, Finland; 10 School of Physics, University College Dublin, Belfield, Dublin 4, Ireland; 11 School of Biology and Environmental Science, University College Dublin, Belfield, Dublin 4, Ireland; 12 Present Address: School of Life Sciences, University of Sussex, Brighton, BN1 9QG UK

**Keywords:** Biophysics, Structural biology, Biophotonics

## Abstract

Soft X-ray tomography (SXT) enables native-contrast three-dimensional (3D) imaging of fully hydrated, cryogenically preserved biological samples, revealing ultrastructural details without the need for staining, embedding, or sectioning. Traditionally available only at synchrotron facilities, recent advances in laser-driven plasma sources have led to the development of compact soft X-ray microscopes. Achieving a resolution of 54 nm full-pitch and tomogram acquisition times of 30 min to two hours, we validate the system across a range of biologically relevant contexts, including protists, yeast, and mammalian cells containing polymeric and inorganic nanoparticles. These use cases establish the robustness of the laboratory based system for studying cell architecture, organelle interactions, and nanoparticle trafficking. By showing that a compact SXT system can achieve reliable high-resolution imaging across various cell types, this study highlights a major step toward making correlative cryogenic X-ray imaging broadly accessible in laboratory settings. Future developments will aim at enhanced throughput, deeper integration with correlative imaging modalities, and extension to more complex specimen types, including tissue.

**Supplementary Information:**

The online version contains supplementary material available at 10.1038/s41598-025-29385-5.

## Introduction

Soft X-ray tomography (SXT) is a three-dimensional (3D) imaging technique that provides native-contrast visualisation of cryogenically preserved fully hydrated biological samples without the need for staining, labelling, sectioning, or embedding. Within the so-called “water window” energy range (between the K-absorption edges of carbon at 284 eV and oxygen at 543 eV), soft X-rays are strongly absorbed by carbon-rich molecules, such as lipids, and only weakly absorbed by water. This enables the detailed imaging of cellular ultrastructure in a near-native state. The technique is equivalent to medical Computed Tomography (CT) performed at the cellular level. Similar to Hounsfield units in medical CT, the data can be quantified based on soft X-ray absorption coefficients^1,2^. Over the past two decades, synchrotron-based SXT has been successfully applied to a broad range of biological questions, including chromatin rearrangement, virus-host interactions, parasite life cycle, cell motility, and nanoparticle trafficking^3^, in addition to applications in materials science. Several dedicated beamlines worldwide (e.g., U41XM at BESSY-II, MISTRAL at ALBA, B24 at Diamond, 24 A at NSRRC, BL07W at NSRL, XM-2 at ALS) have demonstrated the power of the technique for sub-cellular imaging with the spatial resolutions down to lower tens of nanometres as reported in Oton et al.^4^.

Access to SXT has traditionally been restricted to synchrotron facilities, which limits experimental throughput, constrains project timelines, and reduces the feasibility of iterative or exploratory studies. However, advancements in laser-driven plasma sources have led to the development of lab-based microscopes capable of performing SXT. In the recent years, laboratory-based SXT prototypes have been reported^5–9^. These systems, using nitrogen gas puff or liquid nitrogen jet targets for laser-driven plasma generation, have produced high-quality tomograms over reasonable exposure but have not been developed into commercial products due to a combination of engineering and fundamentals challenges^5–9^. Consequently, there has been a lack of a widely accessible, high-performance laboratory instrument capable of bridging the resolution and sample-preservation gap between fluorescence microscopy and electron microscopy.

We address this gap by introducing and demonstrating a laboratory-based soft X-ray microscope that uses a high-brightness laser-driven plasma source with a metal target. For the purpose of this paper, all lab-based SXT data was taken on the microscope developed by SiriusXT. This system achieves resolutions down to 54 nm full-pitch (using same Fourier-ring correlation metric as in Oton et al.^4^) and integrates an epifluorescence microscope to facilitate the development of correlative workflows involving light and electron microscopy^7,10–12^. The question we address is: Can a laboratory-based SXT system deliver sufficient image quality to address the bottleneck in access to the synchrotron-based SXT and enable routine, correlative cryogenic imaging across diverse biological applications?

We present experimental results from multiple use cases – ranging from unicellular protists and yeast to mammalian cells containing nanoparticles – to demonstrate the capabilities of a lab-based SXT. The novelty of our work lies in (i) validating a fully integrated laboratory-based SXT system for high-resolution, 3D cryogenic imaging, (ii) establishing fiducial-free, automated co-registration with fluorescence datasets, and (iii) demonstrating its versatility across a range of biologically relevant specimens. This development represents a significant step towards enabling SXT adoption in core facilities and individual laboratories, and accelerating correlative imaging in cell biology, nanomedicine, and beyond.

## Results

### Lab-based soft X-ray microscope

**Fig. 1:**
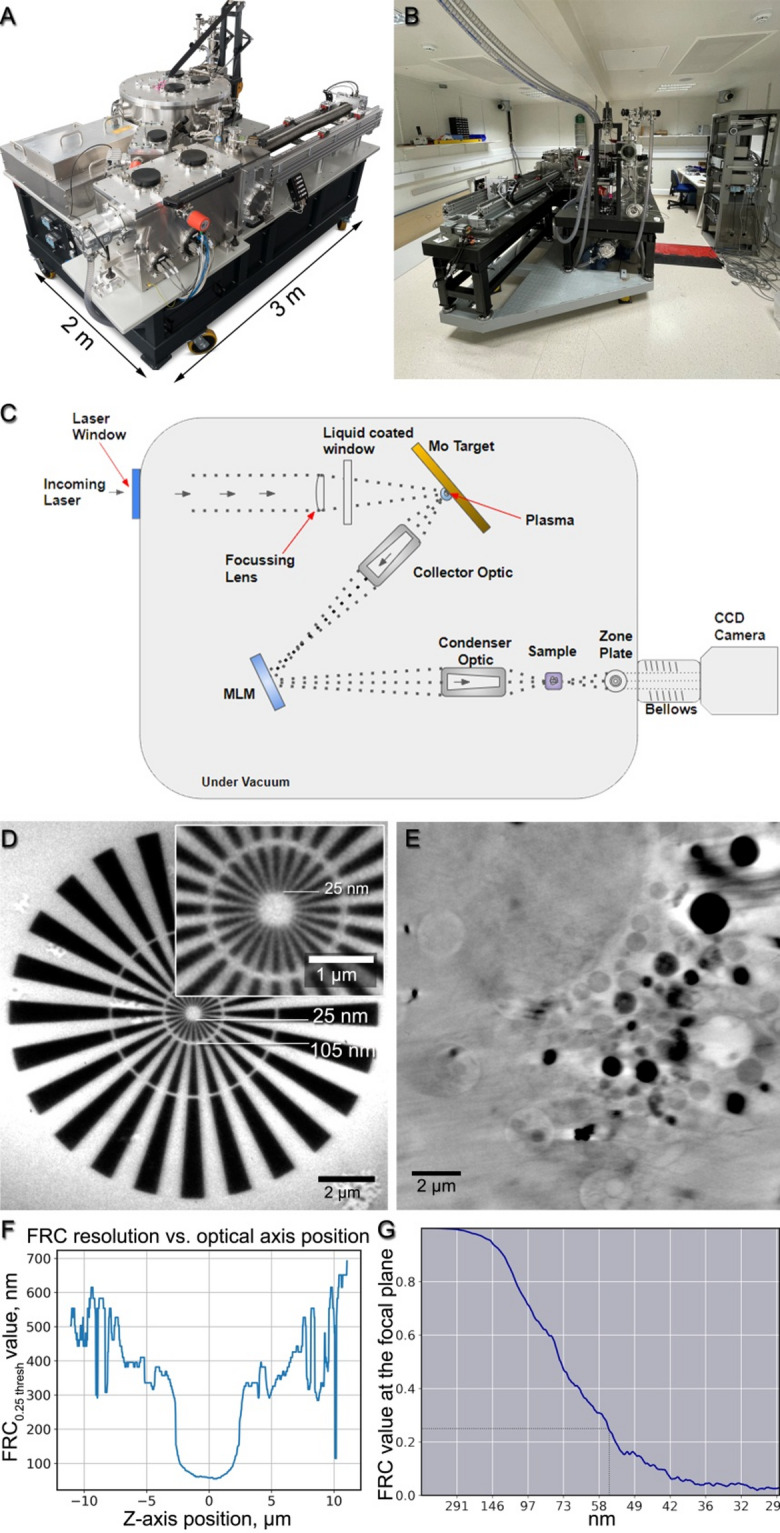
A lab-based soft X-ray microscope, Siemens star and Fourier-ring correlation resolution measurements. (**A**) A lab-based soft X-ray microscope developed by SiriusXT. (**B**) The lab-based microscope in the Conway Institute for Biomolecular and Biomedical Research Imaging Core facility, University College Dublin. (**C**) Optical path diagram. (**D**) A 50-second exposure soft X-ray projection through a Siemens star with the inner ring lines of 25 nm. (**E**) A focal-plane slice from a 3D reconstruction of a mammalian cell tomogram, acquired by the lab-based microscope with a 1.5-hour exposure time over ± 50° tilts, with a 1° tilt step. (**F**) FRC resolution measured at various distances from the focal plane in the 3D reconstruction. (**G**) FRC measurement of the focal plane slice indicating ca. 55 nm resolution. MLM – multilayer mirror, FRC – Fourier-ring correlation.

**Fig. 2:**
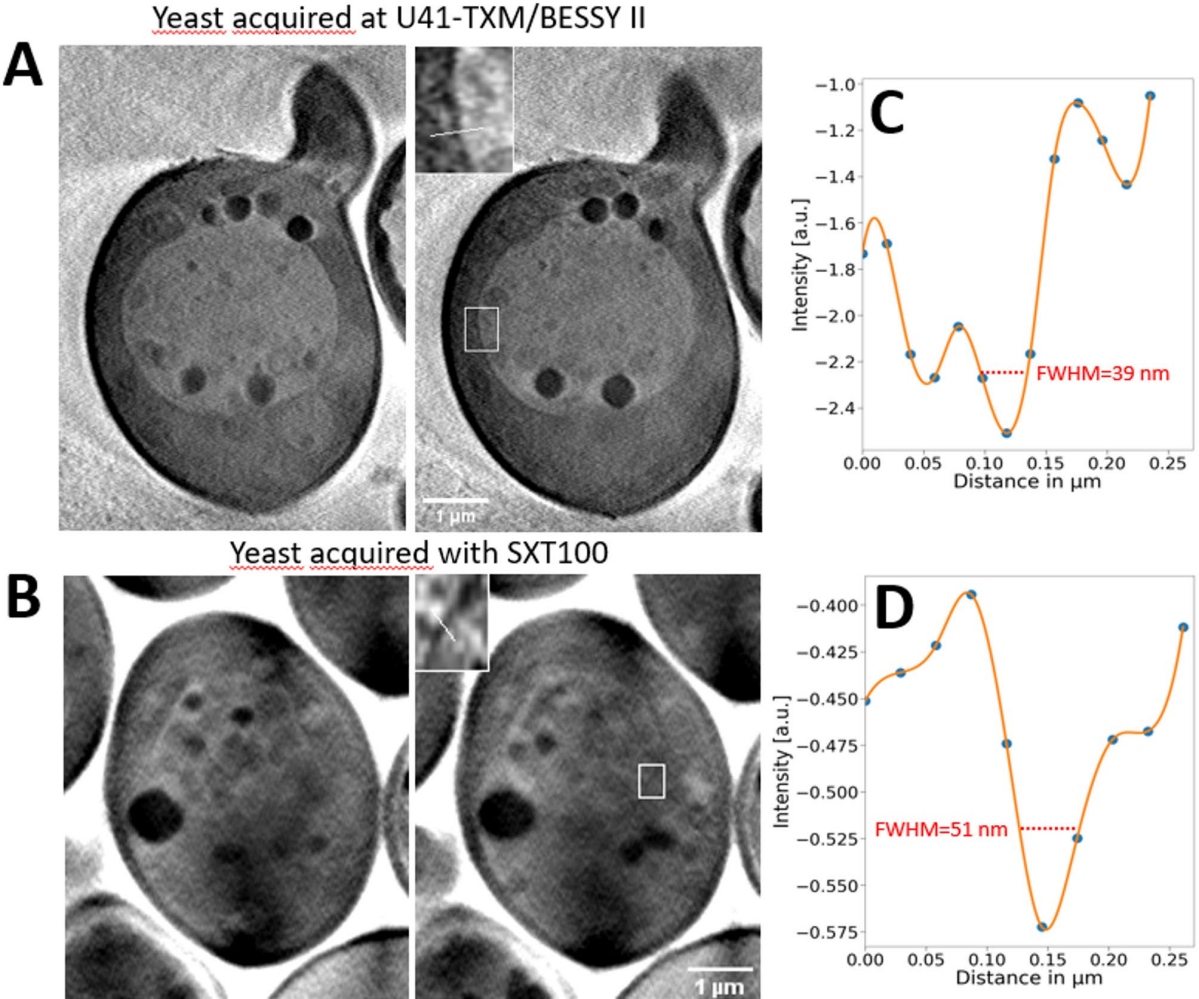
*Saccharomyces cerevisiae* samples prepared identically and imaged in a synchrotron-based and in a lab-based SXT microscope. (**A**) Reconstruction slices through a tomogram acquired at U41-XM beamline at BESSY-II synchrotron, (**B**) Intensity profile and a full width and half maximum (FWHM) measurement across the vacuole membrane in a synchrotron-based tomogram reconstruction slice. (**C**) Reconstruction slices through a tomogram acquired at the lab-based microscope, (**D**) Intensity profile and a FWHM measurement across the vacuole membrane show in a lab-based tomogram reconstruction slice.

The lab-based microscope used in this study (see Fig. 1), achieves 3D resolutions in biological cells down to 54 nm full-pitch, while resolving Siemens star lines and spaces as fine as 25 nm, and enables the efficient acquisition of soft X-ray tomograms of fully vitrified cryogenic samples (Fig. 2). This lab-based soft X-ray microscope features a footprint of 2 × 3 m and includes an integrated cryo-epifluorescence microscope. Typical acquisition times for a single-cell tomogram currently range from 30 min typically for 2–3 μm thick samples to two hours for 5–6 μm, and more for thicker samples. In comparison to other volume imaging techniques that assess the ultrastructure of single cells, such as focused ion beam scanning electron microscopy (FIB-SEM), this allows for high-throughput data for quantitative statistical analysis. While a typical FIB-SEM dataset can resolve structures down to 5 nm, the acquisition time for a single cell at such a resolution can easily be or exceed 48 h, after which the sample is completely destroyed. SXT, on the other hand enables subsequent, targeted high resolution imaging with, e.g., transmission electron microscopy (TEM) or FIB-SEM at cryogenic or room temperatures. The lab-based microscope accepts flat specimen holders, such as standard electron microscopy grids, and cylindrical specimens, such as thin-walled glass capillaries^13^ or cryo-FIB-SEM milled tissue pillars, for full-tilt tomography. A typical tilt series is acquired in 1-degree steps over a 120-degree sample tilt range for flat specimens or 180-degree tilts for cylindrical specimens, collecting in total 121 or 181 projections, respectively. Each tilt angle exposure time varies from 15 to 60 s. An automated stack alignment routine performs fast and fiducial-free alignment before the 3D volume is reconstructed using standard approaches, e.g. weighted back projection (WBP) or Simultaneous Iterative Reconstruction Technique (SIRT) implemented in tomo3D software developed by Agulleiro et al.^14,15^

To achieve high-resolution imaging of hydrated cells, cryogenic vitrification is necessary^16,17^. This process involves rapidly freezing cells using techniques commonly employed in cryo-electron microscopy (cryo-EM), such as plunging cells into liquid ethane cooled by liquid nitrogen^18–20^. No cryoprotectants are required, simplifying sample preparation. However, it is crucial to maintain cells at cryogenic temperatures after freezing to preserve their structure and prevent ice crystallisation or thawing. A cryogenic sample environment has therefore been fully implemented in the lab based microscope^21,22^.

### Resolution measurements

The spatial resolution achieved by the lab-based soft X-ray microscope was evaluated through two distinct methodologies: direct imaging of a Siemens star featuring 25 nm fine structure and Fourier-ring correlation analysis of a tomographic reconstruction of a Huh7 hepatocyte-derived carcinoma cell.

A Siemens star is a standard optical resolution test target, featuring a pattern of spoke-like structures that decrease in size towards the centre of the target. In imaging the Siemens star, lines as small as 25 nm in width were successfully resolved, as shown in Fig. 1. 

For Fourier ring correlation analysis (FRC)^23^, we conducted tomographic scans of a Huh7 cell employing multiple frames per tilt. Subsequently, the frames were divided into two noise-independent datasets following the Tomogram Acquisition scheme outlined in the Methods section. FRC measurements were then conducted for series of reconstruction slices cut parallel to the detector plane, as illustrated in Fig. 1E–G. The FRC threshold value of 0.25 was selected to be consistent with a previously published optical characterisation of the ALBA synchrotron-based soft X-ray microscope at the MISTRAL beamline^4^.

Utilising a threshold set at 0.25 of the correlation values, roughly corresponding to half the signal-to-noise ratio of the full dataset and chosen to maintain consistency with previously published measurements for a synchrotron-based SXT (as detailed in Oton et al. study)^4^, we attained a full-pitch resolution of 54 nm at the focal plane, as depicted in Fig. 1E, F. Furthermore, the depth of field was estimated to be approximately 4.5–5 μm, as shown in Fig. 1F.

A comparison of *Saccharomyces cerevisiae* samples prepared identically and imaged using the lab based microscope and U41-XM beamline at BESSY-II synchrotron is shown in Fig. 2. Here, a measurement of intensity profile across yeast vacuole membrane in both tomograms was made and full width at half maximum (FWHM) calculated. The FWHM value in the lab-based system tomogram was 51 nm, which is consistent with the FRC estimate. The resolution of the synchrotron-based microscope is predictably better, showing FWHM of 39 nm.

### Missing wedge in flat specimens

SXT imaging of samples prepared on flat specimen carriers, such as standard electron microscopy grids, encounters the so-called missing wedge problem similarly to the case of cryo electron tomography (cryo-ET). The missing wedge problem, broadly encountered in cryo-ET and SXT communities, results from a range of sample tilts inaccessible for imaging due to the specimen carrier occluding the view in certain orientations (Fig. S1)^24–26^. This results in missing wedge artefacts manifesting in loss of contrast within the orientations corresponding to the missing information (Fig. S2).

**Fig. 3:**
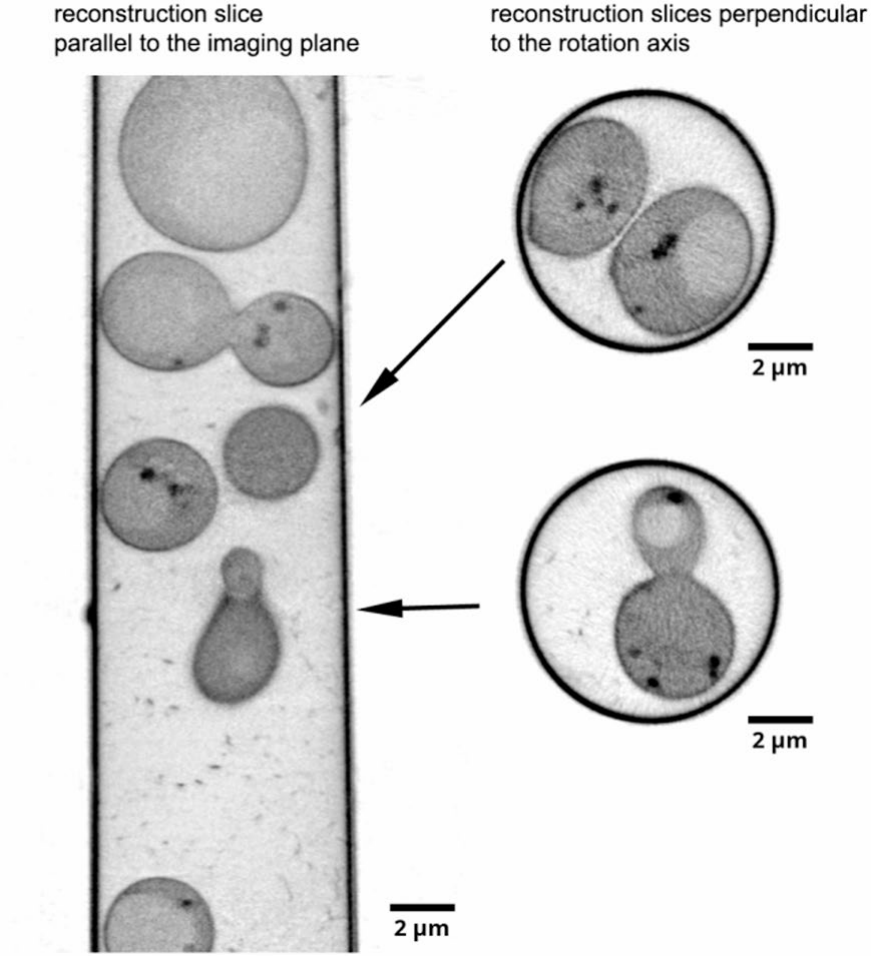
Full-tilt soft X-ray tomography of *S. cerevisiae* plunge frozen in a glass capillary and imaged in the lab-based microscope. The soft X-ray tomogram was collected in 1° steps over ± 90° sample tilt range.

One way to address the missing wedge problem is to implement dual-axis tomography by, e.g., acquiring two tilt series of the same region having rotated the specimen holder by 90° along the optical axis between the tomograms thereby minimising the missing wedge (see Fig. S1b)^27,28^. An alternative approach addressing the missing wedge problem would be employing cylindrical geometry specimens, such as thin-walled pulled glass capillaries, or high pressure frozen specimen/tissue milled to a pillar held from the bottom^3,13,29^. This would provide missing wedge-free full tilt tomography as shown in Fig. S2. Figure 3 shows a full-tilt tomographic reconstruction of *S. cerevisiae* plunge-frozen in a glass capillary imaged using the lab-based soft X-ray microscope. In this work, we will focus on samples prepared on standard 3 mm grids used in electron microscopy bearing in mind presence of the missing wedge in reconstructed tomograms.

### Correlative imaging workflow and freely available imaging routine for the quick co-registration of SXT and epifluorescent datasets of the same cell

The spatial resolution of soft X-ray imaging makes it highly suitable for correlative workflows, bridging the resolution gap between fluorescence and electron microscopy, while capable of providing a native-contrast 3D structural context for the studied regions of interest, without causing EM resolvable damage to the sample (manuscript accepted for publication in Communications Biology)^11^.

The typical workflow^30^ for grid screening and tomogram acquisition starts with a mosaic scan of the grid using an integrated epi-fluorescence microscope. This step provides a visible light overview of the grid, overlaid with fluorescence signals, allowing for a quick assessment of grid quality and cell locations. Next, low-magnification X-ray transmission images, either single or in a mosaic, are acquired to identify regions of interest (ROI) for subsequent tomography acquisition.

Additionally, a low-magnification X-ray projection mosaic is used to find regions of interest for imaging with cryo-volume electron microscopy (cryo-vEM), cryo-ET or other imaging techniques. A 2D grid mosaic of a 1mm^2^ area can be achieved in 20 min. The acquisition times for tomograms typically range from 30 to 120 min. 2D projections alone, acquired over 3–15 s, can typically deliver the necessary information for targeting cells and cell areas for electron microscopy.

**Fig. 4:**
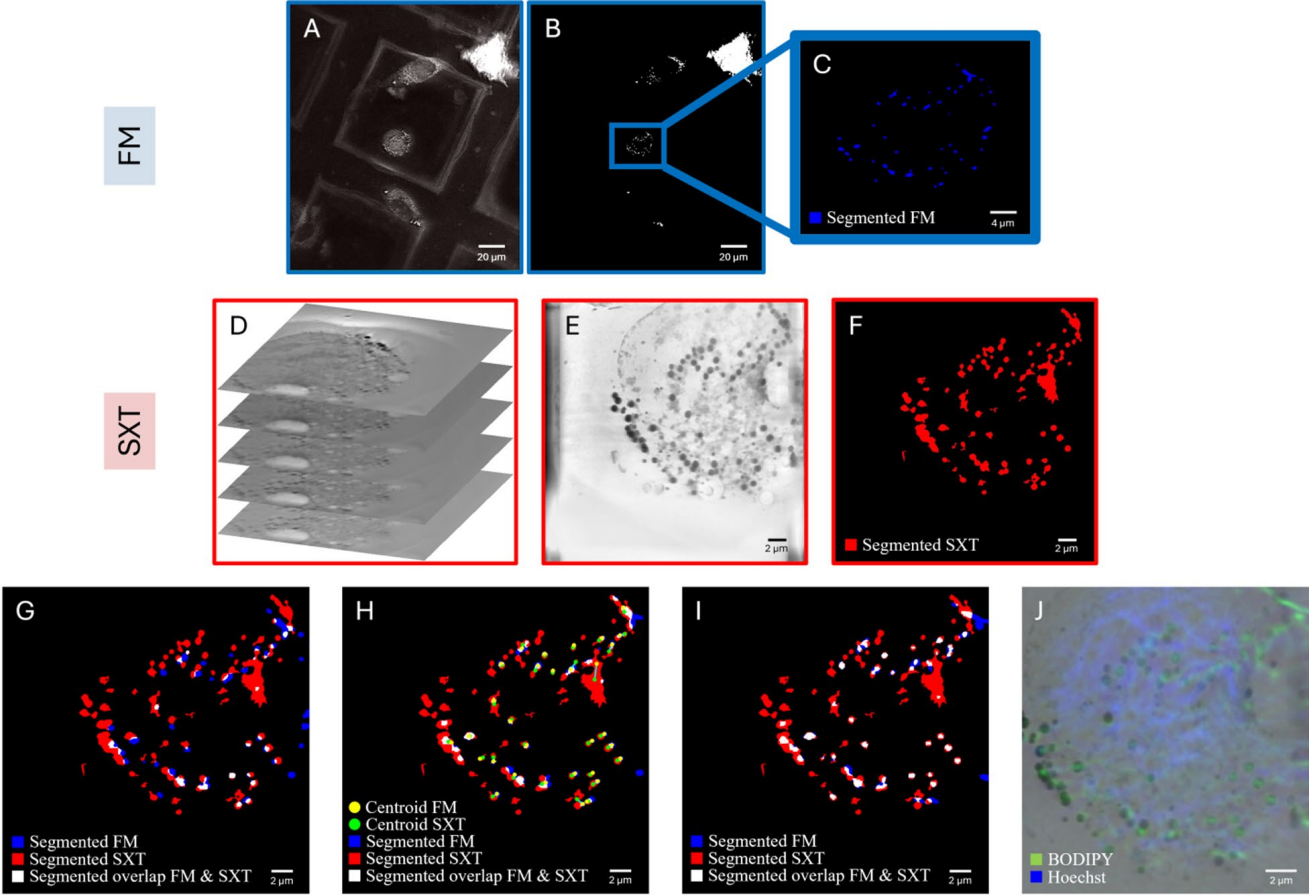
Illustration of the co-registration workflow without the addition of fiducial markers, based on endogenous lipid droplets, visible in both the SXT and wide field fluorescence channel (BODIPY staining – see Materials and Methods for vendor information). The red channel represents the X-ray image, the blue channel corresponds to the fluorescence image. Areas of overlap between the two modalities are displayed in white, indicating regions where both signals coincide. (**A**) Gaussian- and CLAHE-filtered wide field FM image. (**B**) Segmented lipid droplets of the FM modality based on global Otsu’s thresholding. (**C**) Selected Region of Interest to co-register with the SXT dataset. (**D**) Original SXT image stack with the unprocessed data. (**E**) 2D-projected and filtered SXT stack. (**F**) Threshold and size-based segmented lipid droplets. (**G**) The Phase Cross-Correlation (PCC) provides a first, coarse alignment between the X-ray (red) and fluorescence (blue) images. This step serves as the starting point for the alignment process. (**H**) Iterative Alignment with a Cost Function. Yellow points represent the centroids of the red segments, showing their calculated centres of masses. Each yellow point is connected to the corresponding blue segment that it aligns with, demonstrating the progress of the alignment process and the matched pairs between the two modalities. (**I**) The Final Warped Image is achieved using Radial Basis Function (RBF) interpolation. This method addresses local distortions to enhance the correspondence between the X-ray and fluorescence images, resulting in a more precise overlap of the two modalities. (**J**) Overlaid and co-registered minimum intensity projection of the SXT stack and the wide field FM image.

We have furthermore established a workflow that allows for the automated co-registration of the correlative imaging datasets for a single cell, acquired using both SXT and the epifluorescence microscope. The workflow is based on lipid droplets that are easily detectable in both fluorescence and SXT imaging channels and does not require the addition of fiducial markers: For fluorescence microscopy (FM), lipid droplets are stained with BODIPY 493/503, while in SXT, the contrast arises due to their carbon-rich content. Following the automatic detection and segmentation of lipid droplets in both channels based on Gaussian and contrast limited adaptive histogram equalisation (CLAHE) filtering, thresholding and morphological filtering, a phase cross correlation is calculated as a first, coarse alignment step between the two modalities^31^. This is followed by an iterative optimisation based on a cost function and the final co-registration based on a Radial Basis Function (RBF) (see Materials and Methods and Fig. 4). The accuracy of the workflow is evaluated using the leave-one-out approach as published previously^32,33^. Using lipid droplets as natural fiducial markers allows for an accuracy of the co-registration that is well within the pixel size of the low-resolution modality (for the wide field fluorescence microscope of about 500 nm) since the lipid droplet radii range from a few hundred nanometres up to a micron. Furthermore, using lipid droplets instead of artificially added fiducial markers, comes with the advantage that these subcellular structures are endogenous to the cells and distributed throughout them.

The effect of the missing wedge on two-dimensional (2D) co-registration of epi-fluorescence and SXT datasets is negligible. This is because registration is performed using re-projections along the optical axis, parallel to the imaging plane, and can even be achieved using zero-tilt projections alone. This is possible due to the good visibility of lipid droplets in the tilt series.

### Soft X-ray and cryo-light fluorescence imaging of *Euglena gracilis*

**Fig. 5:**
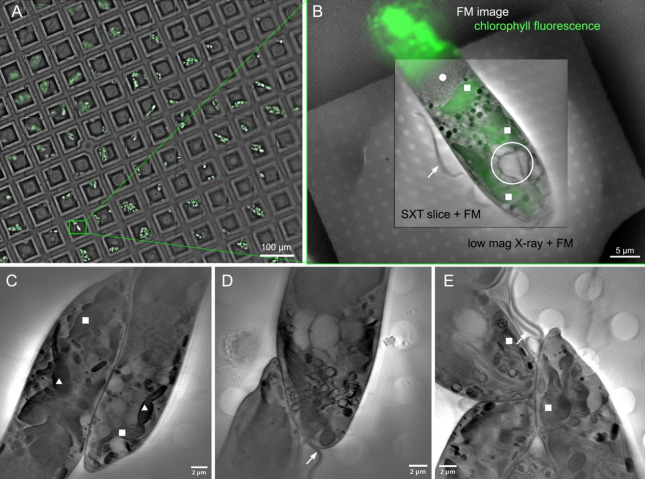
*Euglena gracilis* cells cryo-vitrified on 3 mm Quantifoil-coated TEM grids and imaged by cryo-SXT and the integrated fluorescence microscope in the SiriusXT table-top SXT microscope. (**A**) A visible light fluorescence mosaic of a portion of a 3 mm TEM grid showing grid windows and the chlorophyll fluorescence signal. The area where the soft X-ray tomogram was collected, as shown in panel B, is enclosed in a green rectangle. (**B**) A composite FM image of the *E. gracilis* chloroplast overlaid with a low magnification X-ray projection image acquired with 10 s exposure and a virtual slice through a high magnification SXT tomogram acquired over 1.5 h. (**C**–**E**) Virtual slices through tomograms with 29 × 29 µm^2^ field of view featuring seven *E. gracilis* cells.

A correlative light fluorescence and X-ray images of *Euglena gracilis* cells plunge frozen on a TEM grid are shown in Fig. 5. *E. gracilis* is a complex eukaryotic cell that requires effective ROI targeting of structures for higher resolution cryo-vEM and/or cryo-ET. Correlating ultrastructural information from the entire cell in FIB-SEM with the SXT reconstruction enhances our understanding of the contrast and appearance of organelles and structures in soft X-ray tomograms. Furthermore, the soft X-ray tomograms are highly informative for correlative studies because they can probe the whole vitrified cell volume without the optical distortion or scattering limitations imposed by ice in light and electron microscopies. In the soft X-ray tomograms shown in Fig. 5, absorption based contrast clearly discriminated major organelles. Clearly present were dorsal flagella (arrows), the combined structures containing the feeding apparatus and eyespot, including the ventral flagellum, ventral root, paraxial swelling and flagella pocket vestibulum (white open circle). Integrated fluorescence imaging was able to localise fluorescence associated with photosynthetic systems (chlorophyll fluorescence emission). When combined with the SXT volumes, chloroplasts with thylakoid structures (white squares) and non-fluorescent paramylon granules (white triangles) could be readily identified. Additionally, the nucleus could be clearly located in SXT images (white circle). This information can be employed to target sub cellular and potentially sub-organelle regions of interest for further higher resolution study by EM techniques.

### Soft X-ray tomography of cryo-vitrified yeast cells

**Fig. 6:**
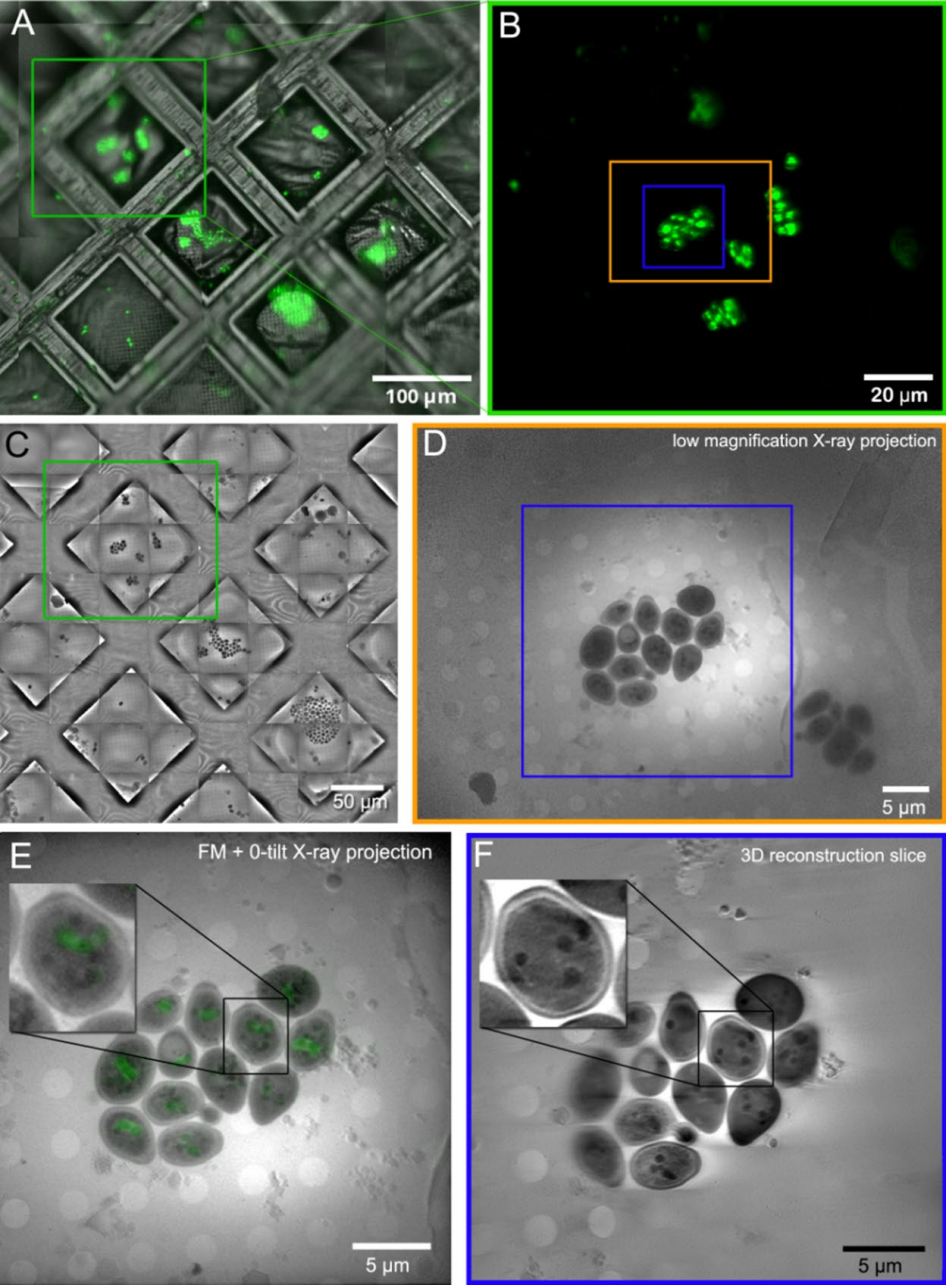
*Saccharomyces cerevisiae* yeast cells imaged using the lab-based microscope. (**A**) A portion of a visible light FM images mosaic of a 3 mm TEM grid with vitrified yeast cells labelled with BODIPY493/503 dye. (**B**) a single fluorescence image of a grid area containing a number of yeast cells. (**C**) A mosaic of low magnification X-ray transmission images covering an area of 370 × 350 µm^2^ acquired in less than 5 min. (**D**) A single low magnification X-ray transmission image capturing yeast cells within a 43 × 58 μm² field of view on a grid window acquired with a 10-second exposure. (**E**) Zero-tilt X-ray tomogram projection displaying a 30 × 30 μm field of view overlaid with a MSSR-processed fluorescence image. (**F**) A virtual slice extracted from the reconstructed tomogram acquired over one hour.

The low magnification X-ray images reveal *Saccharomyces cerevisiae* yeast cells (Fig. 6) as distinct dark elongated objects, approximately 5 μm in size, set against the backdrop of the recognizable pattern of Quantifoil film holes. An example of a combined X-ray and light fluorescence dataset is presented in Fig. 6. A cropped section of an FM mosaic containing a few grid areas where cells were located is shown in Fig. 6A. A specific grid area with visible cells was chosen (green rectangle) and an FM image was acquired (Fig. 6B) followed by a mosaic of low magnification X-ray projections (Fig. 6C) or single low magnification X-ray projections (Fig. 6D) to identify ROIs for a tomogram acquisition. One such ROI is marked with a blue rectangle (Fig. 6D). A zero-degree tilt high magnification projection overlaid with a MSSR-processed^34^ FM image is shown in Fig. 6E, and a virtual slice, extracted from the three-dimensional (3D) reconstructed tomogram with the voxel size of 29 nm is shown in Fig. 6F.

Within yeast cells, discernible submicron-sized dark spherical objects, visible both in X-ray transmission images and the reconstruction were observed. These correspond to lipid bodies, demonstrating pronounced X-ray absorption due to their high carbon content. By contrast, the least absorbing features within yeast cells were vacuoles, primarily containing digestive enzymes in solution. The ability of the lab-based microscope to change magnification and the field of view size not only facilitates screening of the sample (Fig. 6A-C), but also allows high-quality correlation of the necessary lower resolution of epifluorescence with X-ray images to be achieved (Fig. 6E).

**Fig. 7:**
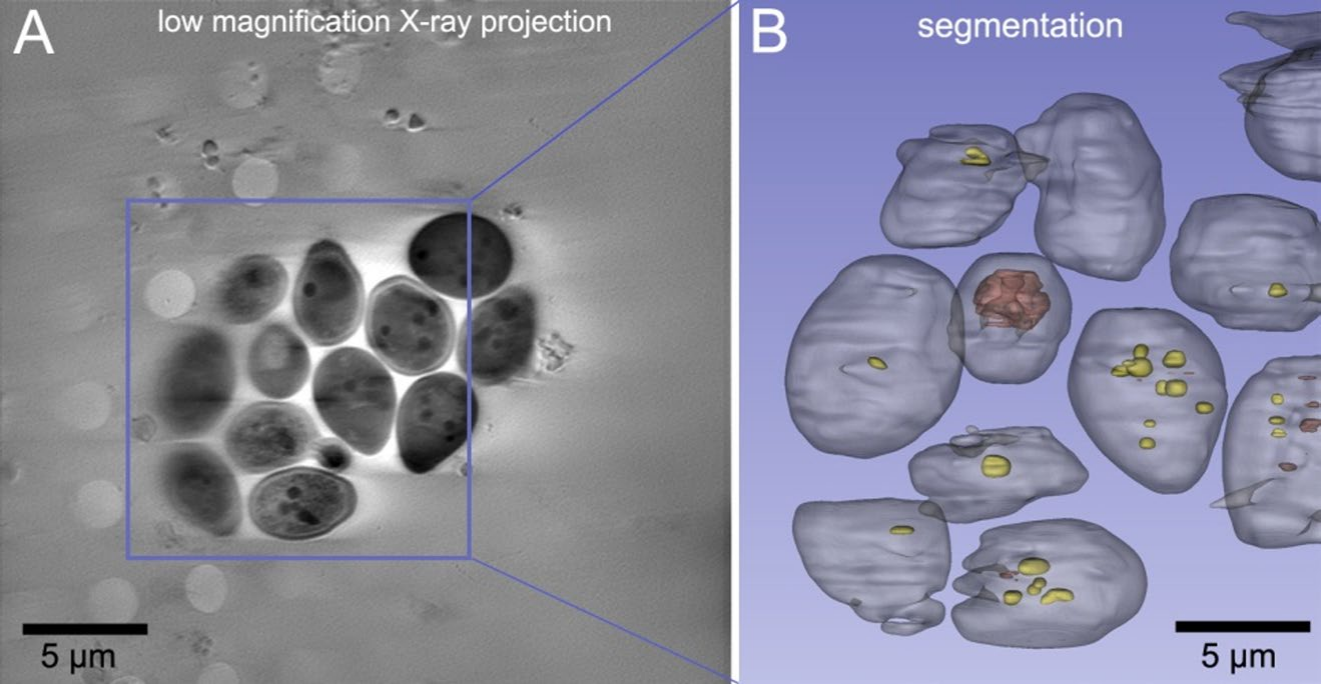
(**A**) A virtual slice extracted from the reconstructed tomogram featuring twelve *Saccharomyces cerevisiae* yeast cells. (**B**) Segmentation surface rendering of a cropped reconstruction region outlined with a blue rectangle. Yeast cells are rendered in grey, lipid droplets in yellow, and digestive vacuoles in brown.

The 3D reconstruction of a high-resolution tomogram (Fig. 7) revealed the spatial arrangement of the droplets relative to the vacuole, enabling study of the process of vacuolar ingestion of lipid bodies during starvation, namely lipophagy^35^.

Yeast vacuoles in cells lacking NCR1/NPC2 lipid transporters appear fragmented or multivesicular in fluorescence images. However, the low resolution and 2D nature of widefield FM images prevents us from discerning the multivesicular ultrastructure. Here SXT perfectly complements the FM data (see Fig. 4D and E in Egebjerg et al.^35^) by offering high resolution 3D ultrastructural information and enables us to confirm internal organisation of yeast vacuoles. Importantly, SXT reveals lipid- or membrane-rich material within the vacuole that does not stain with lipid droplet dyes and only shows faint fluorescence for the membrane marker FM4-64, highlighting the features that fluorescence alone cannot resolve. Furthermore, segmentation of organelles in SXT enables accurate volumetric measurements, which are difficult to obtain with widefield or even confocal FM due to the asymmetry of the PSF and the limited z-resolution (along the optical axis). On the other hand, FM imaging remains necessary for unequivocal determination of organelle identity, which cannot be always achievable by morphological imaging alone using SXT. Together, these imaging modalities complement each other by providing chemical identity of organelles by FM and how these organelles are organised in native cellular ultrastructural context by SXT.

### Imaging nanoparticles in cells using fluorescence and soft X-ray tomography

#### Polymeric nanoparticles in HeLa cells

**Fig. 8:**
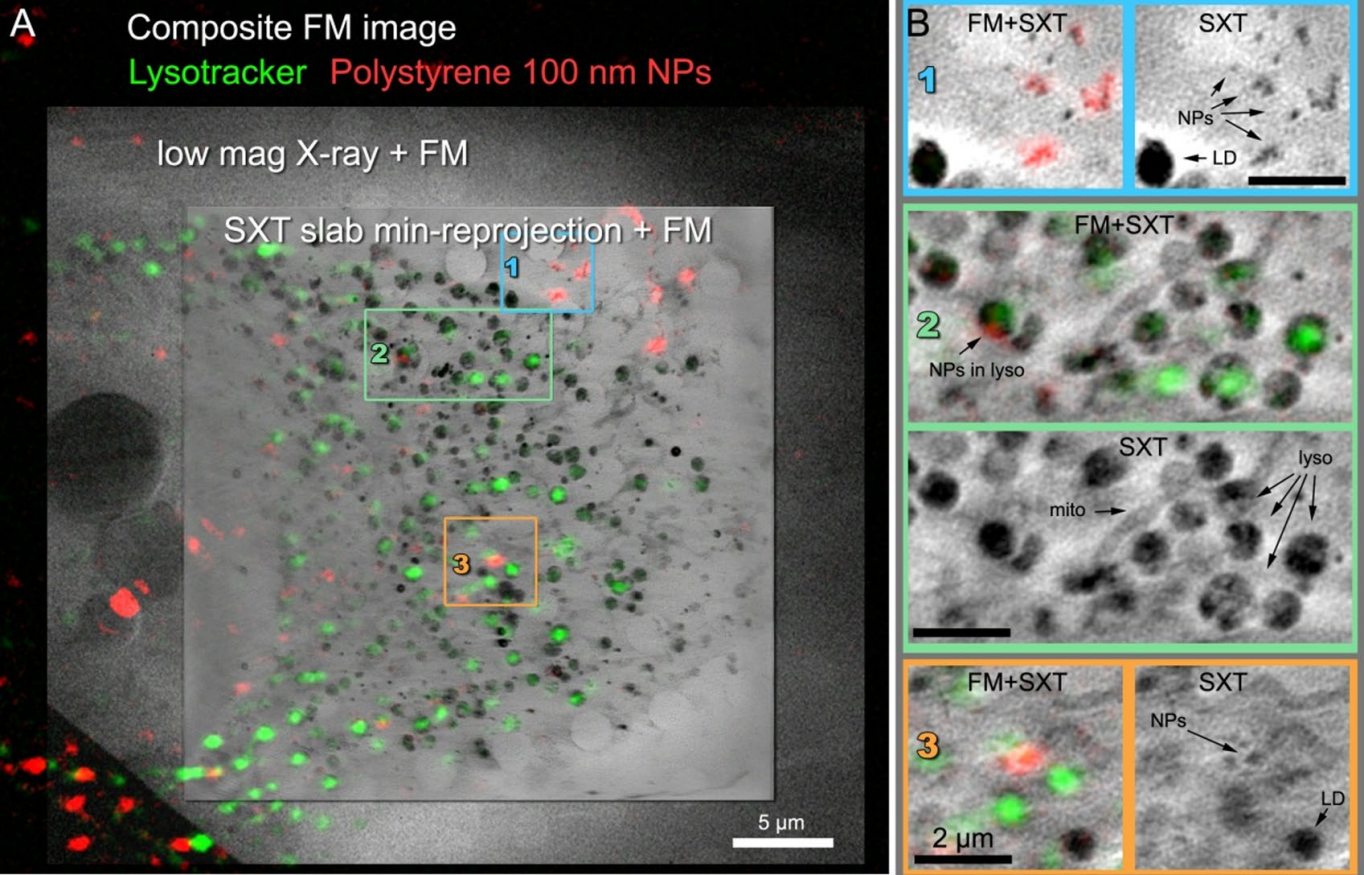
A HeLa cell incubated for four hours with 100 nm fluorescent polystyrene nanoparticles. (**A**) Composite FM image of Lysotracker and fluorescent nanoparticles overlaid with a low magnification X-ray projection and a three high magnification SXT slices across the cell, (**B**) Zoom-in of selected areas in the cell cytoplasm showing the SXT slice (top of each panel) and the same SXT slice overlaid with the composite FM image (bottom of each panel).

Given the ability of the lab-based microscope to resolve subcellular structures in yeast cells, we next applied the system to a use case in cultured mammalian cells. Nanoparticles (NPs) are emerging as important therapeutic delivery vehicles, and gaining a greater understanding of their distribution in the subcellular environment is of high importance to improve their performance. As they are relatively well-characterised in the literature, 100 nm red Carboxylate-Modified FluoSpheres and HeLa cells (Fig. 8A) were used as a model. From previous work using fluorescence light microscopy approaches, these NPs are known to accumulate in lysosomes over time^36^. HeLa cells were pulsed with NPs for 4 h, incubated with Hoechst 33342 (to identify cell nuclei) and LysoTracker (to identify lysosomes), and plunge frozen for cryo-soft X-ray imaging. Red 100 nm FluoSpheres (Fig. 8 B1) were detected at the perimeter of the HeLa cell, this was confirmed by correlating the SXT with the FM image. This also showcased the ability of the lab-based soft X-ray microscope to resolve 100 nm particles. We observed that the FluoSpheres (red) accumulated in lysosomes (green) (Fig. 8 B2), which was consistent with previous studies^36^. In Fig. 8 B2 and B3 mitochondria and lipid droplet morphology can be clearly discerned; this is made possible by exploiting the strong absorption of soft X-rays by carbon dense cellular moieties and is in agreement with that observed in transmission electron micrographs^37^. SXT enables cells to be imaged in their whole, native state, which allows the capture of rare events in the mesoscale to create a more complete picture of how NPs traverse cells when correlated with light microscopy. Understanding the intracellular trafficking of NPs is critical to ensure effective drug delivery when designing novel nanomedicines^36,37^.

#### Inorganic-organic hybrid nanoparticles for cancer therapy

**Fig. 9:**
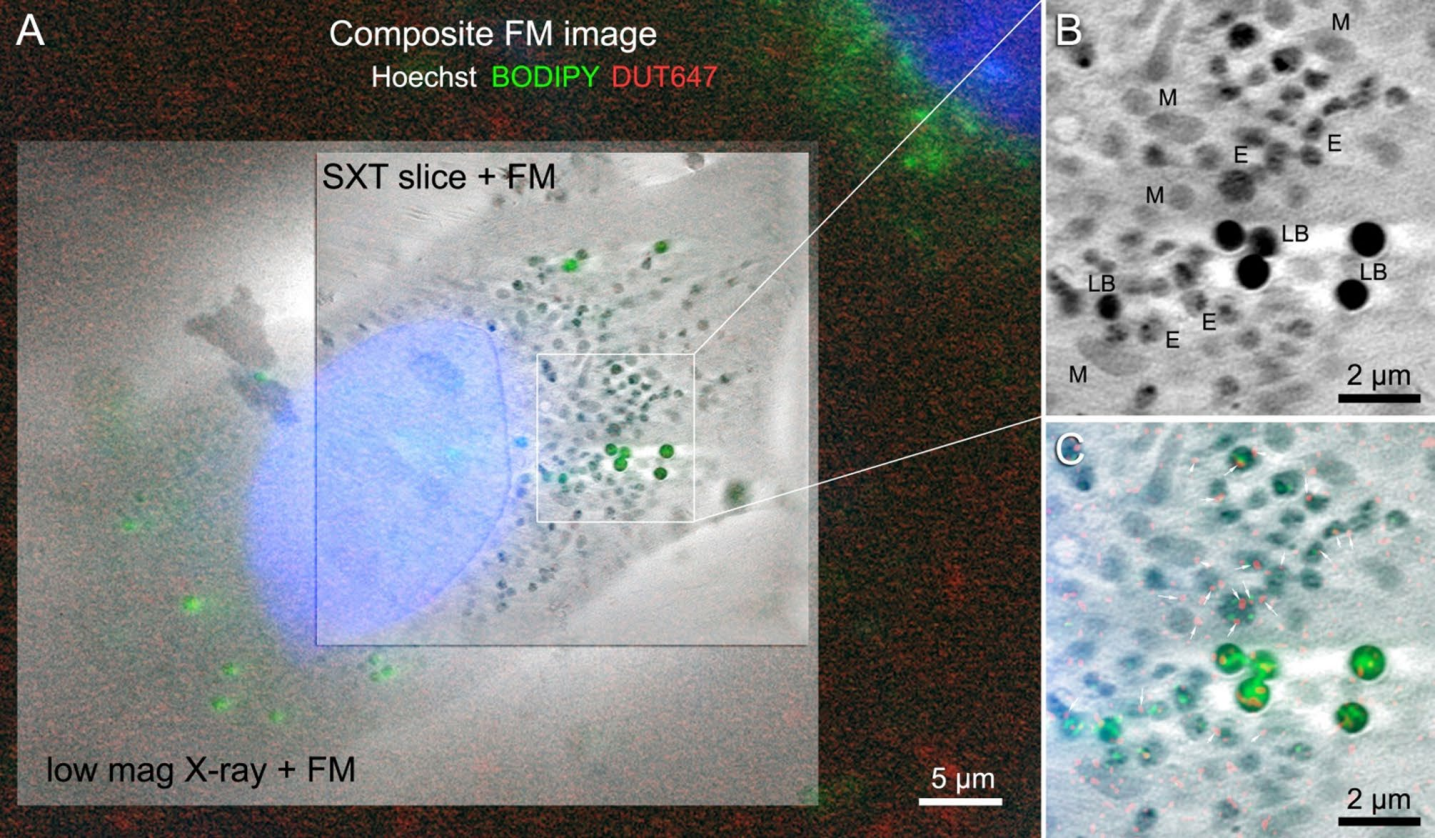
An H8N8 cell incubated for four hours with 50–60 nm [ZrO]^2+^[(CMP)_0.99_(DUT647)_0.01_]^2−^]^2^ IOH-NPs. (**A**) Composite FM image of Hoechst 33342, BODIPY, and DUT647 overlaid with a low magnification X-ray projection acquired over 10 s and virtual slice through a high magnification soft X-ray tomogram acquired over 1.5 h. (**B**) Zoom of a selected area in the cell cytoplasm showing the SXT slice. Abbreviations: LB - lipid bodies, M - mitochondria, E – endosomes. The appearance of most of the endosomes resembles that of lysosomes shown in (Fig. 8). (**C**) Zoom of the same cytoplasm area showing the SXT slice overlaid with the composite FM image. White arrows point to the nanoparticles visible as red dots. The location of the nanoparticles coincides primarily with lysosomes, while a few nanoparticles co-locate with lipid bodies.

Nanoparticles exist in a variety of sizes and materials for therapeutic delivery. Another example is zirconyl-containing inorganic-organic hybrid nanoparticles (IOH-NPs), previously described by Heck et al.^38^ These IOH-NPs consist of an organic drug/dye anion and an inorganic cation such as zirconyl ([ZrO]^2+^) that together result in nanoparticles being insoluble in water. Owing to their distinct composition, which provides X-ray contrast, and their incorporation of a red fluorophore (DUT647: Dyomics-647-uridine triphosphate), the IOH-NPs were visible and detectable using both SXT and FM. With a diameter of ca. 60 nm, single IOH-NPs were resolvable using SXT. These nanoparticles have specific features and advantages of the fluorescent [ZrO]^2+^[(CMP)_0.99_(DUT647)_0.01_]^2−^]^2^ IOH-NPs^−^ (CMP: cytidine monophosphate), that were originally developed at the Karlsruhe Institute of Technology, are: *i*) synthesis in water, *ii*) high load of drug anion and/or fluorescent dye anion (up to 80 wt-% of total nanoparticle mass; here with CMP representing a potential drug anion and DUT647 as fluorescent dye anion), *iii*) high photostability, *iv*) intense emission, and *v*) flexible nanoparticle composition and fluorescence. The therapeutic benefits of the high drug load of IOH-NPs in minimising undesired side effects and overcoming mechanisms of chemoresistance have been demonstrated in a pancreatic cancer mouse model with gemcitabine monophosphate (instead of CMP)^39^. Due to fluorescence labelling, IOH-NPs facilitate monitoring in vivo and ex vivo IOH-NP distribution, confirming their accumulation within tumour tissue and their uptake in tumour cells via endocytosis, followed by intracellular trafficking to late endosomes/lysosomes^39^. To understand the fundamental concepts of how the IOH-NPs interact with the biological systems at cellular and subcellular levels, how physicochemical properties influence intracellular uptake and trafficking and how this might affect nanodrug efficacy, there is a high need to explore the intracellular internalisation, transport of IOH-NP and drug release at a single-cell level. As shown in Fig. 9, this can be achieved by means of correlative fluorescence and soft X-ray-based imaging. To study the internalisation dynamics, murine triple-negative breast cancer cells (H8N8)^40^ were incubated with the IOH-NPs and fixed at 4 h after internalisation. SXT provided sufficient resolution to *(i)* assess the cellular organelles of each cell for each time point and characterise ultrastructural changes that were induced by the IOH-NPs, and to *(ii)* follow the IOH-NP uptake, fate, and co-localisation with organelles (such as lysosomes). Importantly, in contrast to higher-resolution imaging modalities, such as volume EM, SXT was able to provide throughput of 20–30 cell tomograms, and hundreds of low magnification projections per week, to visualise enough cells for each time point to quantitatively and statistically analyse the NP distribution and ultrastructural changes in a reasonable time frame of approximately 2 weeks.

#### Summary

Key findings across the mentioned applications, including measurement parameters are summarised in (Table 1).

**Table 1:** Comparison of Resolution, acquisition Time, and key findings across applications.

Application	Features resolved	Acquisition time	Key findings
Euglena gracilis	~ 54 nm (full-pitch) resolution	~ 1.5 h tomogram	Clear distinction between organelles (chloroplasts, paramylon granules, nucleus, flagella); correlation with chlorophyll fluorescence for targeted cryo-ET/EM.
Saccharomyces cerevisiae (yeast)	~ 54 nm (FRC); 51 nm FWHM	~ 1 h tomogram (29 nm voxel size)	Identification of lipid droplets and vacuoles in 3D; revealed vacuolar lipophagy and multivesicular organisation; quantitative volumetric segmentation feasible.
HeLa cells with 100 nm polymeric nanoparticles (FluoSpheres)	Resolves ~ 100 nm NPs within cytoplasm	~ 1–1.5 h tomogram	Nanoparticles localised to lysosomes; SXT resolved lysosomes, mitochondria, and lipid droplets; correlated FM confirmed NP localisation.
H8N8 breast cancer cells with 50–60 nm zirconyl IOH-NPs	Single NPs (~ 60 nm) resolved	~ 1.5 h tomogram; throughput ~ 20–30 cells in 2 weeks	NP uptake and trafficking to lysosomes; enabled statistical analysis of organelle changes across many cells; demonstrated compatibility with quantitative workflows.
General performance (Siemens star + yeast vs. synchrotron)	25 nm Siemens star; yeast FWHM 51 nm (lab) vs. 39 nm (synchrotron)	30 min (thin cells) – 2 h (thick cells)	High resolution with larger fields of view (up to 45 × 60 μm²) and routine correlative FM-SXT workflows.

## Discussion

The development of a compact SXT microscope, which enables fast high-resolution imaging of fully hydrated, cryogenically preserved biological cells in a laboratory setting, represents a significant advancement in the field of SXT with broad implications for cellular biology. By making SXT accessible to a wide range of researchers at the local lab or core facility level, independent of synchrotron facilities, this microscope opens new avenues for in-depth studies of cell structure across various research fields, as well as for correlative approaches utilising light, X-ray, and/or electron microscopy.

For this study, we used a lab-based SXT microscope produced by SiriusXT. Other noteworthy lab-based SXT microscopes come from research groups at TU Berlin^7,9,41^, KTH Royal Institute of Technology^5,41^ and Jena University^7^. These systems, being research lab-based systems and method-development platforms, rather than a commercial off-the-shelf instrument, show comparable resolutions of tens of nanometres, successful imaging of biological samples, while demonstrating alternative X-ray generation approach. To the best of our knowledge, to date, the microscopes from KTH and TU Berlin also offer cryogenic sample environment^5,8,41^.

Our results demonstrate that the lab-based system achieves high spatial resolutions (see comparison with synchrotron-based SXT above), specifically resolving structures as fine as 25 nm with the Siemens star and achieving a full-pitch resolution of 54 nm using Fourier-ring correlation analysis of reconstructed tomogram slices of biological samples. However, the lab-based microscope currently requires 30 min to two hours to acquire a tomogram, compared to the 5–15 min required by synchrotron-based systems. On the other hand, the lab-based microscope offers a larger field of view, up to approximately 45 × 60 µm^2^, due to its different illumination scheme. Specifically, typical synchrotron-based SXT illumination spot size at the sample is in the order of 1 μm, which is then rastered over a field of 10–15 μm. Conversely, the spot size in the lab-based microscope is a Gaussian with the full width at half maximum of ca. 15 μm (see SI Fig. S4A), which allows imaging up to ca. 45 × 60 µm^2^ (see SI Fig. S4B). Furthermore, synchrotron-based systems can perform full-field absorption spectroscopy allowing for flexible changes in X-ray energy, while the laser-based source of the lab-based microscope source provides a broad spectrum of radiation (see SI Fig. S3). This broad spectrum coupled with available multilayer mirrors allows selection of energies at 398 eV, 453 eV, and 510 eV (see Fig. S3), with 453 eV chosen as the default.

In addition to its high spatial resolution, the compact soft X-ray microscope offers several benefits for cellular and subcellular studies.

One of the primary advantages of SXT is its ability to provide native-contrast 3D structural imaging of whole cells in their close-to-native environment^42^. The microscope’s capability to handle cryogenically vitrified samples at or below − 160 °C through all imaging and sample transfer steps prevents ice crystallisation,^18,21^ maintaining the integrity of vitrified biological samples. This enables visualisation of cellular organelles and ultrastructure without the need for staining or labelling. An integrated epifluorescence microscope further enhances this capability by enabling FM mapping of the specimen without removing the sample from the stage and facilitates correlative light and soft X-ray microscopy (CLXM) – a powerful approach that combines the molecular specificity of FM with the label-free, three-dimensional ultrastructural imaging capabilities of SXT^7,10,12,30,43^.

While FM pinpoints the localisation and dynamics of specific proteins or nucleic acids, SXT provides high-resolution 3D reconstructions of intact, fully hydrated cells without the need for staining or sectioning. By integrating these modalities, CLXM enables the precise mapping of fluorescently tagged molecules onto the native architecture of whole cells, revealing how biomolecules are organized within organelles, membranes, and other ultrastructural features. This synergy bridges the gap between molecular identity and native cell morphology, offering unique insights into cellular organisation and dynamics that cannot be achieved by either technique alone.

For instance, CLXM allows researchers to localise signalling proteins or complexes within specific membrane microdomains or organelle sub-compartments that are not resolved by FM alone. It has been used to study how viral particles, nanoparticles, or drug delivery vectors interact with the native ultrastructure of cells, and to reveal links between gene expression (via FM reporters) and structural remodelling or metabolic state (via SXT density contrast). In virology specifically, fluorescently tagged viral proteins can be tracked during infection, but FM alone cannot determine whether puncta correspond to plasma membrane sites, vesicles, or other compartments. SXT, by contrast, reveals the complete cellular architecture – including plasma membrane topology, organelles, and vesicles – but lacks molecular specificity. Overlaying FM with SXT resolves this ambiguity, enabling precise identification of the ultrastructural sites where viruses assemble and bud^10,44–46^.

The recent advancement of the laboratory-based soft X-ray microscope is accelerating the development of novel correlative imaging protocols. This instrument, owing to its flexibility and daily availability, can be implemented in higher biosafety level laboratories and allow the exploration of new data acquisition strategies, thereby expanding biomedical research that benefits from correlative workflows^46^. Furthermore, the laboratory SXT instrument can be coupled with cryo-ET. We propose a workflow that combines FM, SXT, and cryo-ET to provide complementary information across multiple size scales and levels of biological complexity. In particular, cryo-SXT can enhance information content and targeting accuracy in in-situ structural biology workflows, especially for cryo-ET samples where fluorescent labelling is challenging—such as human pathological material or environmental samples. This integrated approach is the subject of a separate manuscript currently in preparation.

Given the ability of SXT to generate a 3D map of entire cells, the technique is well-suited to capture rare events (e.g. nanoparticle endosomal escape), which might be missed when slicing through small portions of a cell by EM^47^. The ability to visualise entire cells in 3D with native contrast allows researchers to identify and analyse these rare events within their full cellular context, which might otherwise be missed or overlooked using other imaging techniques. Importantly, within a week, almost 50 cells and different time points can be imaged. For instance, in our study of 100 nm nanoparticle-fed HeLa cells, we demonstrated the utility of this integrated system by combining soft X-ray tomograms with fluorescence images, allowing for precise localisation of nanoparticle markers within the cellular context. Similarly, in the study of [ZrO]^2+^[(CMP)_0.99_(DUT647)_0.01_]^2−^]^2^ IOH-NP distributions within H8N8 breast cancer cells, the ability to visualise the distribution and impact of nanoparticles on cellular morphology helps in understanding the intracellular uptake and transport mechanisms of nanoparticle-based drug delivery systems to treat cancer cells. Dynamic studies over time will be able to assess not only the trafficking but also the degradation process of the IOH-NPs.

The ability of SXT to locate cells or specific areas under the sample’s ice coating, combined with providing a reliable estimate of ice thickness and quality, adds another layer of utility for sample preparation and quality control in cryo-EM studies. Given that the radiation dose from an SXT tomogram typically ranges from 4 to 100 MGy (see Groen et al.^11^ and SI for calculations), equivalent to < 1 to 20 cryo-ET projections, depending on chosen exposure and sample thickness, SXT offers a minimally invasive approach that complements downstream imaging^11^. The radiation dose limit for sample imaging by electron microscopy is considered around 550 MGy^11,48,49^.

Another important aspect of SXT is the quantitative nature of its absorption contrast, which follows the Beer-Lambert law^1,16^. This enables the distinction of cellular structures or quantification of concentrations by measuring linear absorption coefficients from SXT reconstructions. This approach works particularly well for cylindrical sample geometries, such as thin glass capillaries loaded with cells^50–52^, which are also supported by the lab-based microscope^30^.

Overall, the ability to resolve fine structures with high-throughput, combined with its native-contrast quantitative imaging capabilities^53,54^ and its role in facilitating complementary imaging techniques, underscores the versatility of compact SXT systems and their strong potential for advancing research on cellular structure and function.

### Integration and limitations

####  Dataset registration

The registration of the FM and SXT datasets based on their features enabled the reliable mapping of fluorescently labelled lipid droplets to their structural context. By rescaling the FM data, applying robust filtering, and executing a coarse-to-fine alignment workflow, we achieved pixel-level registration accuracy (mean error ~ 622 ± 123) nm. This finding indicates that lipid droplets can function as effective endogenous markers for multimodal image registration. A similar approach had been suggested by Scher et al. to correlate 3D cryogenic datasets between fluorescence and focused ion beam scanning electron microscopy^55^. The advantage of using lipid droplets as fiducial markers lies in their endogenous origin. In contrast, externally introduced markers such as nanobeads generally remain associated with the cell membrane and therefore offer only limited spatial coverage of the intracellular volume. Lipid droplets, by comparison, are intrinsic to the cells and distributed throughout the cytoplasm, which facilitates reliable correlation across imaging modalities while reducing the risk of artefacts that may arise from external artificial markers. Although lipid droplets have been demonstrated to serve as reliable endogenous fiducials, their morphological variability limits the universal applicability of this approach across different cell types and experimental conditions. The use of exogenous spherical fiducial markers can improve consistency, yet it also entails additional steps in sample preparation. Nevertheless, the automated registration pipeline constitutes a robust replacement for previous manual alignment procedures in image editing software, enabling reproducibility and batch processing of datasets.

####  Missing wedge

Another limitation inherent to flat specimen carriers is that some tilt angles are inaccessible due to the carrier blocking X-ray transmission. This results in a missing wedge of information and corresponding reconstruction artefacts along those directions. The missing wedge can be reduced by acquiring two tomograms of the same region, rotating the specimen by 90° between acquisitions (Fig. S1)^27^. Alternatively, using cylindrical carriers such as pulled glass capillaries (see Fig. 4) or FIB-milled pillars from high-pressure frozen samples, can eliminate the missing wedge altogether.

####  Ice contamination and poor vitrification

In cryo-SXT, ice formation can arise from several sources and compromise image quality. This issue has been extensively covered in the context of SXT imaging in previous publications^8,19,30,56^. During sample preparation, incomplete vitrification due to insufficient cooling rates or excessive sample thickness. In storage and handling, temperature fluctuations above the devitrification threshold or brief exposure to humid air introduce frost contamination, while poor cryogen management increases risk. During transfer, condensation of atmospheric moisture on grids is a common cause of ice contamination, especially if using an inefficient cryo shuttle. Beam-induced warming, if not addressed, can also result in devitrification. Together, these factors highlight that ice originates from incomplete vitrification, devitrification, or frost contamination, each tied to vulnerabilities in preparation, transfer, or imaging workflows.

To counteract the above risks, the lab instrument comes with a dedicated LN2 (liquid nitrogen) sample transfer station which protects the sample from atmospheric contamination during loading. Transfer is done within a cooled transfer stick and held inside a shroud within its own atmosphere of N_2_ and loaded to the microscope via vacuum load lock. Repeated transfers of the same sample revealed minimal contamination accumulation. Within the microscope vacuum chamber itself, the sample is surrounded by a thermal shroud which provides radiative cooling and minimises vapour contamination. Furthermore, the sample is docked to the cryo stage cooled via copper braids connected to a cryostat controlled by temperature sensors attached to the sample stage providing continuous feedback further removing warming and avoiding ice devitrification.

The integrated FM allows for fast screening of grids and can quickly identify cases of strong ice contamination or cases of very thick vitreous ice. In addition, an area of the grid can be screened in soft X-ray projection mode to rapidly identify cells of interest. These 2D projections are usually sufficient to identify cells that look healthy and are well vitrified, before proceeding to a 3D tomogram^12^. By way of further validation, samples have been imaged in the lab instrument and then transferred to cryo-FIB for lamella preparation before cryo-ET, resulting in excellent quality data (work in progress).

*Data collection throughput.* The main limitation of this study was the restricted number of cells imaged, limiting statistical power. Sample preparation also remains a bottleneck, as ice contamination, thickness variability, and cell condition at vitrification are difficult to control. Improved correlation with SXT will require higher-resolution fluorescence microscopy, achievable with higher NA objectives or advanced methods such as cryo-SOFI or cryo-SIM, though additional transfers for offline imaging carry risks of contamination^43,57–59^. Looking forward, automated segmentation will accelerate the identification and quantification of subcellular features, nanoparticles, etc., while automated screening and batch tomogram acquisition with multi-grid holders will streamline cell selection and imaging. Together, these advances will increase throughput, improve reproducibility, and enable more comprehensive analysis of cell structure and heterogeneity across the biological context.

### Future directions

The use of a compact laser-based metal target source to generate water-window soft X-rays in a full-field X-ray microscope will bring SXT into labs and core facilities by providing direct and instant access to this imaging modality. On-going development of the lab-based SXT microscope, such as multiple grid handling, biosafety levels 2/3 sample shuttles, tissue slab imaging, and options for confocal, super-resolution fluorescence, and Raman spectroscopy, as well as full automation, will broaden the impact and enhance the throughput of this imaging modality further. As described above, CLXM and cryo-SXT offer the potential for integration into correlative light and electron microscopy (CLEM) workflows owing to the near non-destructive nature of SXT imaging, relatively small size of the microscope, and cryo-vitrified sample handling, all of which are highly compatible with cryo-CLEM workflows.

One particularly promising correlative light, electron, and X-ray microscope (CLEXM) direction for lab based SXT is tissue imaging. Presently, workflows including high pressure freezing followed by cryo-FIB milling and tissue lift outs or cryo-FIB milling of tissue pillars imaged by FM and SXT followed by thinning and cryo-ET are being tested and fine-tuned.

Lab-based SXT also aligns with the growing emphasis on studying cell heterogeneity and individual cell morphology, key areas of focus for large-scale projects such as the Human Cell Atlas and the pancreatic beta cell consortium^60,61^. Its ability to image intact, cryo-preserved cells in three dimensions without the need for fixation, staining, or sectioning makes it invaluable for studying structural variations that influence cell and tissue function, nanodrug delivery and toxicity, and disease progression. This capability is essential for understanding complex biological systems at the single-cell level, a critical aspect of modern cell biology and precision medicine.

Integrating advanced computational methods for image registration, segmentation, and analysis, such as deep learning techniques, will further expand the scope of SXT in biological research. This combination of SXT with machine learning has already proven fruitful for in-depth interpretation of synchrotron-generated X-ray image data of cells and will be a valuable addition to a lab-based SXT system in the near future^35,62,63^.

## Materials and methods

### Yeast sample preparation

BY4741 yeast (source: Euroscarf – Y00000) was incubated at 30 °C in YPD media consisting of 2% D(+)-glucose monohydrate (Merck, 1.08342.1000), 2% Bacto Peptone (BD Chemicals 211,677), 1% yeast extract (Merck, 1.03753.0500), and 0.02% adenine (Sigma-Aldrich, A-2786) for 24 h before spinning down and resuspending the cells in PBS. Lipid droplets were labelled using a final concentration of 12 µM 4,4-difluoro-1,3,5,7,8-pentamethyl-4-bora-3a,4a-diaza-s-Indacene (BODIPY 493/503; Thermo Fisher, D3922) before cells were added to polylysine-coated Quantifoil R2/2 TEM grids (Au G200F1) for plunge freezing and imaging. The grids were plunge frozen on Leica GP2 plunge freezer using 3 s blotting times.

### *Euglena gracilis* sample preparation

*Euglena gracilis* (Culture Collection of Algae and Protozoa (CCAP) Freshwater member of the Euglenophyta: *Euglena gracilis* Klebs CCAP 1224/5Z) was routinely cultured in *E. gracilis* medium and Jaworski’s medium mixed in 1:1 proportions^64^. Cultures were maintained at 15 °C under a 12:12 h light: dark regime. Illumination was provided by cool white LED lamps with a photon flux density of 50 µmol·m^− 2^·s^− 1^ at the surface of the culture vessel. For plunge freezing the cells were pipetted (4 µl) on EM grids (gold/200mesh/R2.2 either UltrAufoil or Quantifoil) in a humidified chamber (98% RH), single side back blotted (20 s) before vitrification by plunging into liquid ethane using an EM GP Plunge Freezer (Leica microsystems, Austria).

### HeLa cell sample preparation

HeLa cells (human cervical cancer cell line, ATCC CCL2) were cultured in Dulbecco’s Modified Eagle Medium (DMEM) (Life Technologies, 31885023) with 10% foetal bovine serum (FBS) (Life Technologies, A5256801) on Quantifoil R2/2 gold mesh EM grids (Jena Biosciences, X-103-Au200), which were glow discharged in an argon/oxygen (75/25) atmosphere for 5 min. Cells were seeded at 10 × 10⁴ density and cultured overnight. Fluosphere 100 nm nanoparticles (Invitrogen, F8784) were added at 50 µg/µl for 4 h to ensure uptake. Nuclei were stained with Hoechst 33342 (Sigma, 62249, 0.2 µg/ml) for 1 h, and lysosomes with LysoTracker (Thermo Fisher, L7526, 1 µM) for 30 min. Cells were washed with PBS and subsequently plunge-frozen using a Leica GP2 with a 6-second blotting time to enable vitrification and avoid ice crystal formation.

### H8N8 cell sample preparation

For correlative fluorescence and SXT imaging, H8N8 mammary carcinoma cells^40^ (see Maenz et al.^65^ for the cell origin) were cultured in DMEM high glucose media (Dulbecco’s Modified Eagle Medium; Roth, 9005.1), supplemented with 10% FBS (foetal bovine serum; Merck, S0615), on Quantifoil SiO2 Au 200 R1.2/20 grids. The grids were prepared by glow discharging in an argon/oxygen atmosphere (75/25) for a period of two minutes, followed by a coating of 50 µg/ml fibronectin for 30 min (Merck, F1141-1MG). This was followed by a washing step with PBS to enhance cell adherence. The cells were then added to the grids and cultured overnight.

[ZrO]^2+^[(CMP)_0.99_(DUT647)_0.01_]^2-^]^2^ IOH-NPs were added to the cells at a concentration of 10 µg/ml and incubated for a period of four hours to ensure sufficient uptake. The lipid droplets were labelled using a final concentration of 5 µM 4,4-difluoro-1,3,5,7,8-pentamethyl-4-bora-3a,4a-diaza-s-indacene (BODIPY 493/503; Thermo Fisher, D3922). Nuclei were labelled using a final concentration of 5 mM Hoechst (Thermo Fisher, 62249). Both stainings were incubated for 30 min. For cryopreservation, plunge freezing on a Leica GP2 with a blotting time of 4 s was performed to achieve vitrification, preventing ice crystal formation and preserving the ultrastructure.

### Image registration using lipid droplets

To integrate the FM and SXT datasets into a common coordinate space, a multi-step registration workflow was implemented. This workflow accounts for the differences in spatial resolution and information content of the two modalities: Initially, the FM images (~ 120 nm/pixel) were rescaled by interpolation and ROI-cropped to align with the physical pixel size of the SXT images (~ 29 nm/pixel). This ensured that both modalities shared the same spatial sampling and physical scale. Subsequently, both the FM and SXT datasets underwent a systematic pre-processing procedure: The application of Gaussian filtering served to reduce noise, while CLAHE was employed to enhance local contrast. Otsu-thresholding was then used to produce clean binary masks of lipid droplets. Secondly, we performed a feature-based registration using lipid droplets as endogenous fiducial markers. The selection of lipid droplets was guided by their consistent visibility in both FM (due to BODIPY staining) and SXT datasets. In SXT, lipid droplets characteristically manifested as circular, ring-like structures, whereas in FM, the detection was confined to the labelled lipid content. Due to the missing 3D FM information, the lipid droplet SXT data was projected into a two-dimensional format prior to the extraction of the centroid to match the corresponding FM images. These centroids were then utilised as corresponding points for the registration process.

A first alignment was conducted using phase cross-correlation (PCC), followed by the iterative optimisation of shifting and scaling to achieve precise overlay based on a cost function (affine transformation). Subsequently, the lipid droplets in both images were determined and aligned using a Radial Basis Function (RBF). Local non-rigid warping was additionally employed to account for distortions between the two imaging modalities (for example due to fried-egg-like shape of plunge-frozen cells resulting in slight lensing effect for visible light) and corrected for local misalignments based on thin-plate spline interpolation. As a final step, we quantified the accuracy of the registration, using the leave-one-out error analysis of the matched lipid droplets centroids based on the protocol published by Kukulski et al. using external fiducial markers^32,33^. Each landmark is omitted from the landmark set in turn, the chosen registration transform is computed from the remaining landmarks, and that transform is used to predict the omitted landmark’s coordinates in the target modality. The prediction error is defined as the Euclidean distance between predicted and measured positions. For each iteration of the leave-one-out validation, we removed the left-out lipid droplet segment only from the second image, while keeping the first (reference) image fixed as the coordinate frame for refitting and error computation. The registration (PCC-based translation followed by cost-driven affine refinement with anisotropic scaling and rotation) was then recomputed from the original unregistered binary images. The excluded segment was subsequently transformed into the reference coordinate space, and its prediction error was quantified as the Euclidean distance between the predicted and actual centroids.

The multi-step registration program is available at https://github.com/Fuchs-L/BODIPY-Overlap-Correlation.git.

## Supplementary Information

Below is the link to the electronic supplementary material.

Supplementary Material 1

## Data Availability

The data that support the findings of this study are available from SiriusXT, Dublin, Ireland but restrictions apply to the availability of these data, which were used under license for the current study, and so are not publicly available. Data are however available from the corresponding author, Sergey Kapishnikov, upon reasonable request and with permission of SiriusXT.
