# Supplementary material for: Demonstrating soft X-ray tomography in the lab for correlative cryogenic biological imaging using X-rays and light microscopy
Source: Sci Rep. 2025 Nov 27;15:45491. doi: 10.1038/s41598-025-29385-5 (PMC12749342; doi:10.1038/s41598-025-29385-5)
Supplement: Supplementary file 1 — Supplementary Material 1 [file 41598_2025_29385_MOESM1_ESM.docx]

**Demonstrating Soft X-Ray Tomography in the lab for correlative cryogenic biological imaging using X-rays and light microscopy**

Stephen O’Connor^1,a^, David Rogers^1,a^, Maryna Kobylynska^2,3^, James A. Geraets^3,b^, Katja Thaysen^4^, Jacob Marcus Egebjerg^4^, Madeleen C. Brink^5^, Louisa Herbsleb^6^, Michaela Salakova^6^, Leon Fuchs^6^, Frauke Alves^7^, Claus Feldmann^8^, Axel Ekman^9^, Paul Sheridan^1^, William Fyans^1^, Tony McEnroe^1^, Fergal O’Reilly^10,1,11^, Kenneth Fahy^1^, Roland A. Fleck^2,3^, Daniel Wüstner^4^, Jeremy C. Simpson^5^, Andreas Walter^6^, Sergey Kapishnikov^1,11,*^

^1^ SiriusXT, 9A Holly Ave. Stillorgan Industrial Park, Blackrock, Co, Dublin, Ireland

^2^ Randall Centre for Cell and Molecular Biophysics, King’s College London, London, SE1 1UL, United Kingdom

^3^ Centre for Ultrastructural Imaging, King’s College London, London, SE1 1UL, United Kingdom

^4^ Department of Biochemistry and Molecular Biology, University of Southern Denmark, Odense M, Denmark

^5^ Cell Screening Laboratory, School of Biology and Environmental Science, University College Dublin, Belfield, Dublin 4, Ireland

^6^ Center of Optical Technologies, Aalen University, Aalen, Germany

^7^ Max-Planck-Institute of Multidisciplinary Sciences, Translational Molecular Imaging, Göttingen, Germany

^8^ Karlsruhe Institute of Technology (KIT), Institute of Inorganic Chemistry, Karlsruhe, Germany

^9^ Department of Biological and Environmental Science and Nanoscience Centre, University of Jyväskylä, Jyväskylä, Finland

^10^ School of Physics, University College Dublin, Belfield, Dublin 4, Ireland

^11^ School of Biology and Environmental Science, University College Dublin, Belfield, Dublin 4, Ireland

^a^ S.O’C. and D.R contributed equally to this work

^b^ current address: School of Life Sciences, University of Sussex, Brighton, BN1 9QG, United Kingdom

^*^ corresponding author

correspondence: [sergey.kapishnikov@siriusxt.com](mailto:sergey.kapishnikov@siriusxt.com)

**Supporting information**

**Missing wedge in flat specimens**


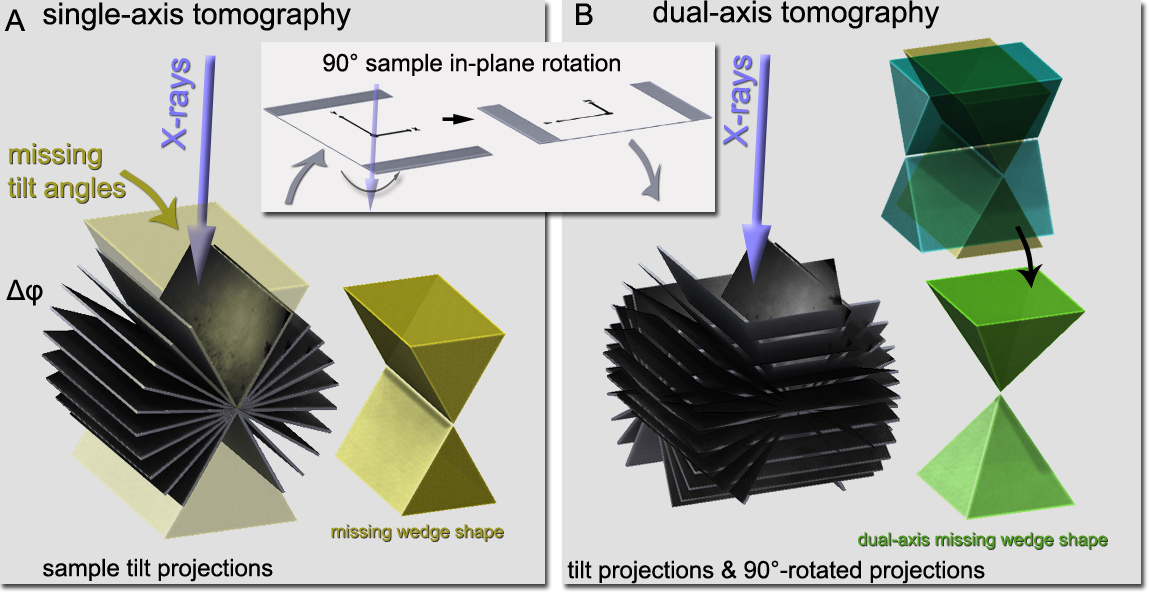


**Fig. S1** Missing wedge shapes in (A) single-axis and (B) dual-axis tomography.


^1^


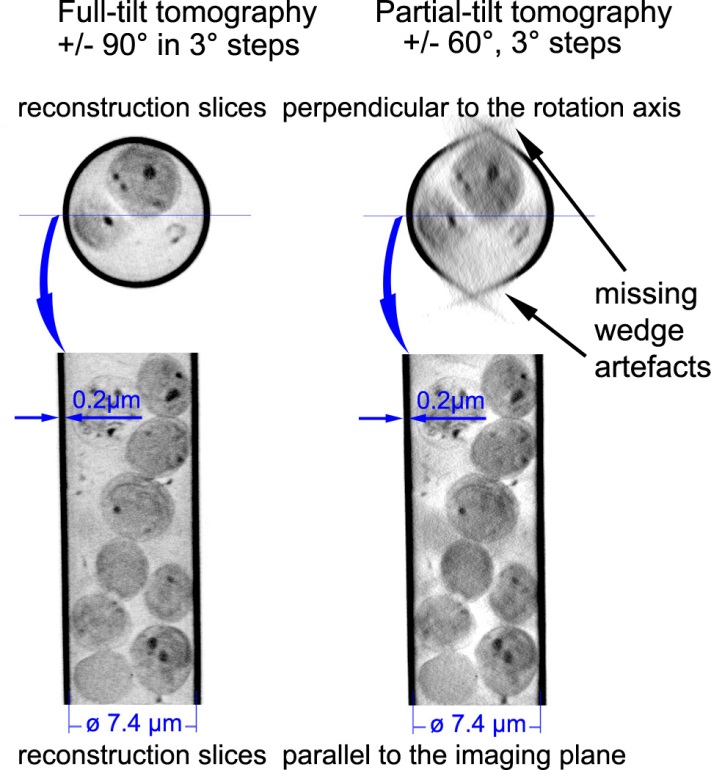


**Fig. S2** Full-tilt (left) and partial tilt (right) tomography. Missing wedge artefacts are clearly visible manifest as loss of contrast and geometric distortion of sections falling within the missing wedge, generally along the optical axis. Within reconstruction slices cut parallel to the imaging plane and the 0-tilt projection, the missing wedge artefacts manifest as a variation in image contrast perpendicular to the rotation axis without visible geometric distortions. This imaging plane is used for 2D correlation of fluorescence and soft X-ray images.


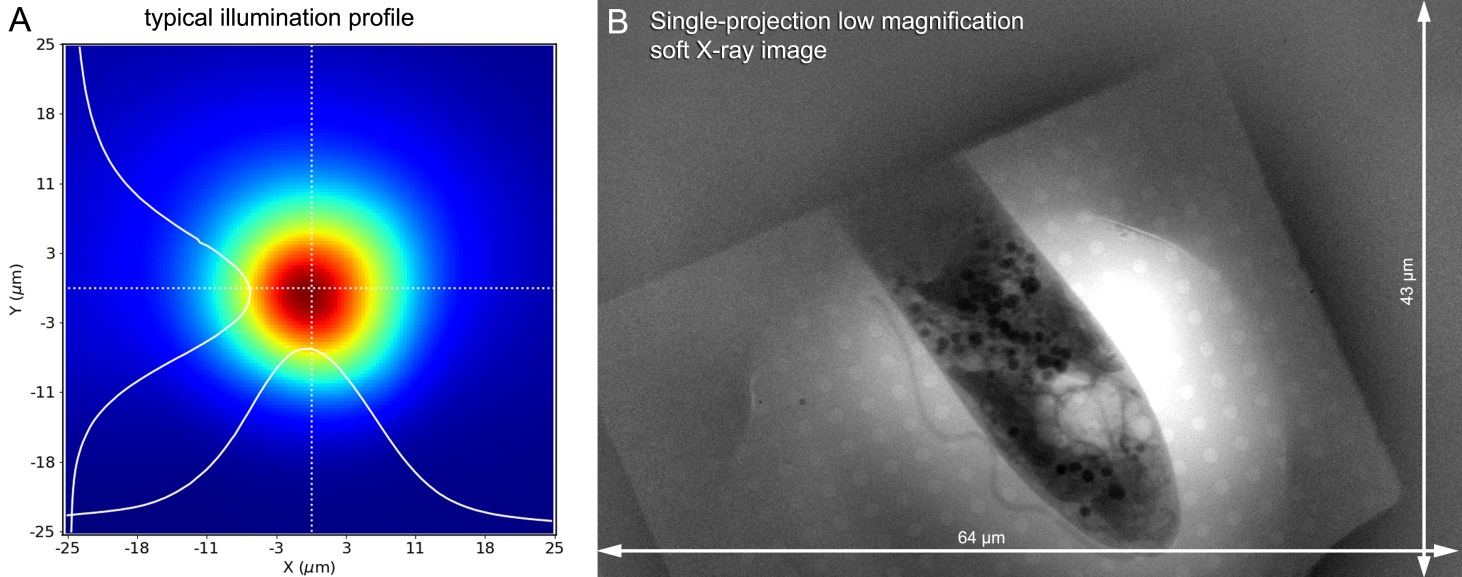


**Fig. S3** Typical illumination profile and a low magnification projection used for finding regions of interest. (A) Typical illumination profile. (B) A 5-second exposure low magnification X-ray projection image of a *Euglena* cell frozen on a TEM grid used for finding regions of interest for tomogram acquisition.


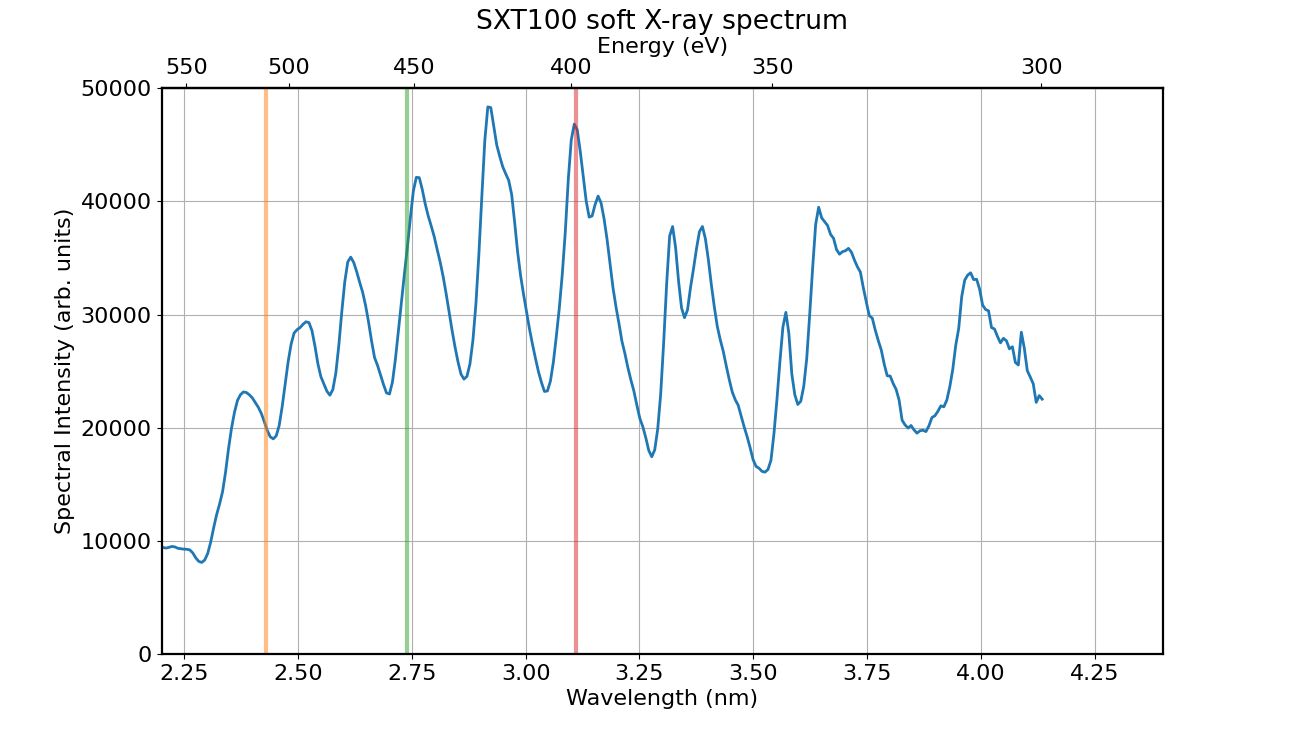


**Fig. S4** Laser-driven plasma source spectral intensity in the soft X-ray region. Commercial multilayer mirrors are available at the wavelength labelled with orange, green, and red lines. The wavelength chosen as the default for imaging is 2.74 nm corresponding to ~453 eV.

**X-ray radiation dose calculation**

The radiation dose of an imaged area is calculated as follows. For every X-ray projection in the tilt series the number of absorbed photons are measured by subtracting transmitted counts from the flat field, and dividing this difference by the detector counts per photon (~29 for 453 eV) and by the Fresnel zone plate objective efficiency (0.16 for the nickel zone plate used). The result is multiplied by the photon energy, ~7.2×10^-17^ J for 453 eV to give the amount of absorbed energy. To convert the result into Gray (Gy), we divide the resulted absorbed energy by sample mass in the scanned area. For this we assume sample density of approximately that of water, 1 g/cm^3^ and obtain sample thickness from the tomogram reconstruction. While we estimate average dose, we recognise that radiation does distribution through the sample, particularly, thick samples, is not uniform with higher radiation dose received by the side facing the source. However, for most applications where we image cells or tissue cryo-lift outs of typically 1-2 micrometers thick and rarely exceed 4 micrometers, which results in transmissions >50% for sample ice and hence the dose distribution difference is insignificant for the task or rough evaluation of the dose suitability for downstream imaging by electron microscopy.


^2^ The estimated dose varying generally between 4 and 6 MGy for shorted exposure tomograms, 10-20 MGy for average tomograms, far below the limiting value of <550 MGy cited in literature.


^3,4^ In rare cases of particularly long tomograms for imaging thick samples (e.g., >10 micrometers) the calculated average doses reach up to 100 MGy. However, we do not use these samples for downstream electron microscopy imaging.

**References**

1. The 3D figure was created using SolidWorks 2021.

2. Groen, J. *et al.* Bridging the resolution gap in cryo-CLEM by introducing cryo-SXT: cryo-CLXEM. *bioRxiv* 2025.09.05.673626 (2025).doi:10.1101/2025.09.05.673626

3. Baker, L. A., Rubinstein, J. L. & Jensen, G. J. Chapter Fifteen - Radiation Damage in Electron Cryomicroscopy. *Cryo-EM Part A Sample Preparation and Data Collection* **481**, 371–388 (2010).

4. Henderson, R. & Clarke, B. C. Cryo-protection of protein crystals against radiation damage in electron and X-ray diffraction. *Proceedings of the Royal Society of London. Series B: Biological Sciences* **241**, 6–8 (1997).
